# Hypersensitivity reactions to small molecule drugs

**DOI:** 10.3389/fimmu.2022.1016730

**Published:** 2022-11-10

**Authors:** Jiayin Han, Chen Pan, Xuan Tang, Qi Li, Yan Zhu, Yushi Zhang, Aihua Liang

**Affiliations:** ^1^ Institute of Chinese Materia Medica, China Academy of Chinese Medical Sciences, Beijing, China; ^2^ Institute of Information on Traditional Chinese Medicine, China Academy of Chinese Medical Sciences, Beijing, China

**Keywords:** drug hypersensitivity reactions, small molecule drugs, allergic drug hypersensitivity reactions, non-allergic drug hypersensitivity reactions, screening hypersensitivity reactions in clinical practice, hypersensitivity evaluation for investigational new drugs or post-marketing drugs

## Abstract

Drug hypersensitivity reactions induced by small molecule drugs encompass a broad spectrum of adverse drug reactions with heterogeneous clinical presentations and mechanisms. These reactions are classified into allergic drug hypersensitivity reactions and non-allergic drug hypersensitivity reactions. At present, the hapten theory, pharmacological interaction with immune receptors (p-i) concept, altered peptide repertoire model, and altered T-cell receptor (TCR) repertoire model have been proposed to explain how small molecule drugs or their metabolites induce allergic drug hypersensitivity reactions. Meanwhile, direct activation of mast cells, provoking the complement system, stimulating or inhibiting inflammatory reaction-related enzymes, accumulating bradykinin, and/or triggering vascular hyperpermeability are considered as the main factors causing non-allergic drug hypersensitivity reactions. To date, many investigations have been performed to explore the underlying mechanisms involved in drug hypersensitivity reactions and to search for predictive and preventive methods in both clinical and non-clinical trials. However, validated methods for predicting and diagnosing hypersensitivity reactions to small molecule drugs and deeper insight into the relevant underlying mechanisms are still limited.

## Introduction

Drug hypersensitivity reactions (DHRs) encompass a broad spectrum of adverse drug reactions (ADRs) with heterogeneous clinical presentations and mechanisms (1). Acute/delayed cutaneous responses, inflammation of the respiratory tract and/or gastrointestinal system, cytokine release syndrome, and/or anaphylaxis are commonly observed in DHRs (2). Many investigations have been performed to explore the underlying mechanisms involved in DHRs and to search for predictive and preventive methods in both clinical and nonclinical trials. A widely accepted dogma is that large molecular weight (>1,000 Da) agents, such as polypeptides, proteins, and polysaccharides, possess immunoreactivity, which prompts them to elicit DHRs directly. Therefore, methods for testing the allergenic potential of these drugs have been relatively developed. In contrast, small molecule drugs (≤1,000 Da) do not have the ability to stimulate immune responses only by themselves. Thus, they trigger hypersensitivity reactions in other ways, which are more complicated and difficult to evaluate accurately (3, 4). To date, validated methods for predicting and diagnosing hypersensitivity reactions to small molecule drugs and deeper insight into the relevant underlying mechanisms are still limited.

## Classification of drug hypersensitivity reactions

ADRs are defined as unintended and noxious reactions to drugs that occur at doses normally used for prophylaxis, diagnosis, or treatment (5). According to the World Health Organization consensus, ADRs are categorized into predictable (type A) and unpredictable (type B) reactions. Predictable ADRs are usually dose dependent and attributed to the known pharmacological or toxic properties of the causative drugs, which can occur in all individuals. The risks for predictable ADRs are illustrated before the clinical application of the drugs (6). Overdose, drug interactions, or off-target side effects of the drugs themselves are the main causes of type A reactions (7). In contrast, unpredictable ADRs generally occur only in predisposed subjects and lack correlation with any pharmacological property of the drugs (8); these unpredictable ADRs make up approximately 10%–15% of all ADRs (9). Inherent host characteristics, such as human leukocyte antigen (HLA) alleles, genetic polymorphisms, metabolism, physical state, illness, and viral infections, as well as factors of drugs including structure, dose, route of treatment, and duration of exposure, influence susceptibility to unpredictable ADRs (10–13). Type B reactions are more difficult to recognize and perceive through preclinical and clinical trials before drug application on the market, and they usually incur large costs. DHRs are unpredictable ADRs that are attributed to off-target stimulation of immune responses or other non-immune pathophysiological processes. DHRs have been estimated to affect more than 7% of the general population and have become an important public health problem in daily clinical practice (14, 15). According to the involved immune system and contributed cells, DHRs are classified into allergic drug hypersensitivity reactions (ADHRs) and non-allergic drug hypersensitivity reactions (NADHRs).

**Figure 1:**
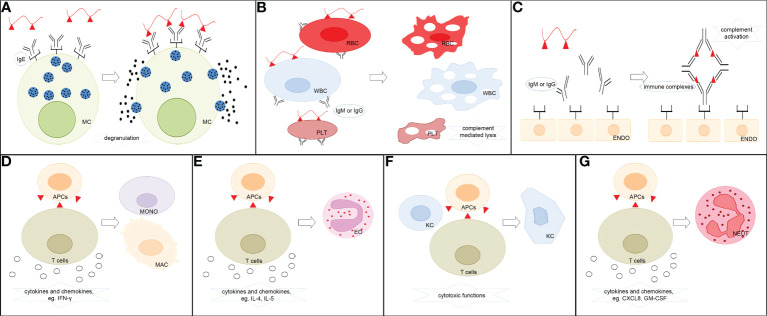
Classification of ADHRs. **(A)** Type I ADHRs. Drugs stimulate the generation of drug-specific IgE, which binds to high-affinity IgE receptors on the surface of mast cells and basophils. The drugs then react with specific IgE–effector cell complexes and induce the release of histamine and other inflammatory mediators. **(B)** Type II ADHRs. Specific IgG or IgM antibody-coated cells encounter complement-dependent cytotoxicity to induce cell lysis. **(C)** Type III ADHRs. Deposition of drug–antibody complexes activates the complement system or other immune cells to induce inflammation and injury. **(D)** Type IVa ADHRs. Th1 cells mediate macrophage/monocyte activation. **(E)** Type IVb ADHRs. Th2 cells mediate eosinophil activation. **(F)** Type IVc ADHRs. Cytotoxic T cells act as effector cells to produce cytotoxic mediators and kill tissue cells. **(G)** Type IVd ADHRs. T cells mediate neutrophil activation. MC, mast cells; RBC, erythrocytes; WBC, leukocytes; PLT, platelets; ENDO, endothelial cells; MONO, monocytes; MAC, macrophages; EO, eosinophils; KC, keratinocytes; NEUT, neutrophils; APCs, antigen-presenting cells.

Currently, ADHRs are categorized primarily based on Gell and Coombs’ classification system into type I reactions (also known as immediate-type allergic reactions) mediated by immunoglobulin E (IgE) antibodies, type II reactions (also known as cytotoxic allergic reactions) elicited by immunoglobulin G (IgG) or M (IgM) antibodies, type III reactions triggered by immune-complex deposition, and type IV reactions (also known as delayed-type reactions) induced by cellular immune mechanisms (16) **(** **Figure 1**). The ADHR classification is listed in **Table 1**. In type I ADHRs, drug-specific IgE antibodies are produced by drug-activated B-lymphocytes after sensitization. Existing IgE antibodies can bind to high-affinity FcRI present on the surface of mast cells and basophils to form IgE–effector cell complexes (sensitized, asymptomatic state). When drugs with the same or similar molecular structures are re-exposed to immunized individuals, they can immediately bind to the cross-linking specific IgE and, within minutes, stimulate the release of inflammatory mediators such as histamine and tryptase, and elicit the subsequent rapid generation of prostaglandins and cytokines, which cause a systemic inflammatory reaction (mainly in the skin and airways) such as urticaria, anaphylaxis, and asthma (3, 14, 17). The most famous small molecule drug that causes type I ADHRs is penicillin. In type II ADHRs, specific IgG or IgM antibodies are produced in response to drugs on the surface of erythrocytes, leukocytes, and/or platelets that induce complement-dependent cellular cytotoxicity reactions or macrophage-related cell clearance. According to the target cells, hemolytic anemia, cytopenia, granulocytopenia, and thrombocytopenia are the most commonly observed reactions. To date, antibiotics, anticonvulsants, sulfonamides, and heparin have been reported to cause type II reactions (3, 9). In type III ADHRs, specific IgG or IgM antibodies bind to the drugs and form immune complexes. The immune complexes load onto host tissues, causing complement fixation to generate C3a and C5a, or bind to other immune cells to release inflammatory mediators and cytokines and increase vascular permeability (19). Vasculitis, arthralgia, serum sickness, and drug fever are associated with type III reactions. Penicillin, sulfonamides, thiouracils, and phenytoin may induce these reactions (11). Different from type I–III reactions that are mediated by antibodies secreted from B cells, type IV ADHRs are induced by activated T cells, which are further sub-categorized into types IVa–IVd according to the dominant cytokines and preferential activation of different immunocytes. Both CD4^+^ and CD8^+^ T cells participate in type IV ADHRs, of which CD8^+^ T cells are considered to play a major role (20). In type IVa ADHRs, Th1 T cells secrete large amounts of interferon (IFN)-γ to activate macrophages/monocytes, which promote the production of complement-fixing antibodies and co-stimulate proinflammatory reactions and CD8^+^ T-cell responses. Therefore, the IVa reactions are commonly combined with the IVc reactions. In type IVb ADHRs, Th2 T cells mainly secrete cytokines IL-4 and IL-5 to induce B cells to produce IgE and IgG4 and trigger eosinophil responses. In type IVc ADHRs, CD4^+^ and CD8^+^ T cells act as effector cells to produce cytotoxic mediators and kill tissue cells (keratinocytes) in a perforin/granzyme B and/or FasL-dependent manner. These reactions, including Stevens–Johnson syndrome (SJS) and toxic epidermal necrolysis (TEN), are the most common severe ADRs in clinical practice. In type IVd ADHRs, T cells activate and recruit neutrophils *via* the secretion of chemokines, such as C-X-C motif chemokine ligand 8 (CXCL8) and granulocyte-macrophage colony-stimulating factor (GM-CSF), to induce sterile neutrophilic inflammation (18, 21, 22). In fact, ADHR categories may overlap in clinical practice. Patients can simultaneously exhibit comprehensive symptoms from multiple types of reactions (4).

**Table 1:** Classification of ADHRs (3, 6, 14, 16–18).

	Type		Cell type	Mechanism	Typical clinical symptoms	General onset time
ADHRs	I		B cells	Drugs stimulate generation of drug-specific IgE, which binds to high-affinity IgE receptors on the surface of mast cells and basophils. The drugs then react to specific IgE–effector cell complexes (predominantly through the cross-linking between IgE and its high-affinity receptor FcRI on the mast cells and basophils) and induce the release of histamine and other inflammatory mediators	Anaphylaxis, urticaria, angioedema, bronchospasm	within 1–6 h, mostly < 1 h
	II		B cells	Specific IgG or IgM antibody-coated cells (predominantly erythrocytes, leukocytes, and platelets) encounter complement-dependent cytotoxicity to induce cell lysis or macrophage clearance	Granulocytopenia, hemolytic anemia, cytopenia, thrombocytopenia	1–14 days
	III		B cells	Deposition of drug–antibody complexes activate the complement system or other immune cells to induce inflammation and injury	Vasculitis, arthralgia, serum sickness, drug fever	2–21 days
	IV	IVa	T cells	Th1 cells mediate macrophage/monocyte activation	eczema	1–28 days
		IVb	T cells	Th2 cells mediate eosinophil activation	Eosinophil-rich maculopapular exanthema, bullous	
		IVc	T cells	Cytotoxic T- cell activation to produce cytotoxic mediators and kill tissue cells	Bullous, maculopapular exanthema (MPE), Stevens–Johnson syndrome (SJS), toxic epidermal necrolysis (TEN)	
		IVd	T cells	T cells mediate neutrophil activation	Pustular exanthema	

DHRs are also classified into immediate and non-immediate/delayed reactions, according to their onset time. Immediate DHRs commonly occur 1–6 h after drug administration, whereas non-immediate DHRs usually occur from more than 1 h to several weeks after eliciting medication (14). Immediate ADHRs are conventionally referred to as type I reactions, which mostly occur within the first hour following the first administration of a new course of drug treatment. Meanwhile, type IV ADHRs, which are mainly caused by T cells and typically occur 48–72 h or even days to weeks after drug exposure, are considered delayed reactions.

NADHRs are also known as pseudo-allergic or anaphylactoid reactions. Typical clinical symptoms observed in NADHRs, including rash, urticaria, angioedema, bronchoconstriction, gastrointestinal signs, and anaphylaxis, are practically identical to those resulting from type I ADHRs, but no immune mechanism has been proven to participate in the reactions (23). Indeed, effector cells correlated with ADHRs, such as mast cells and basophils, usually contribute to NADHRs. In clinical practice, NADHRs may occur in each treatment without prior drug-specific sensitization. These reactions usually arise immediately during or after the first administration of drugs, which is quite distinct from IgE-induced allergic reactions (6, 24). It has been estimated that NADHRs make up two-thirds of immediate DHRs (25, 26).

## Mechanisms of allergic hypersensitivity reactions from small molecule drugs

**Figure 2:**
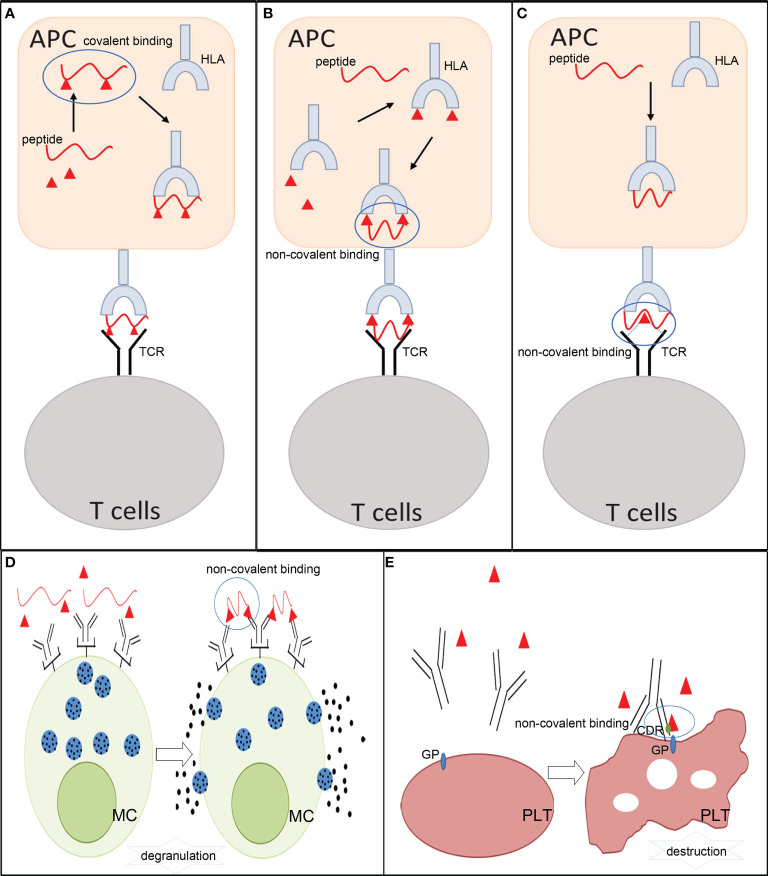
The mechanisms involved in allergic hypersensitivity reactions to small molecule drugs. **(A)** Hapten theory. **(B)** Altered peptide model of p-i HLA and allo-immune model of p-i HLA. **(C)** p-i TCR model. **(D)** Fake antigen model. **(E)** Drug-induced immune thrombocytopenia. APCs, antigen- presenting cells; HLA, human leukocyte antigen; TCR, T-cell receptor; MC, mast cells; PLT, platelets; GP, glycoprotein; CDR, complementary-determining region.

As exogenous substances, small molecule drugs can be hydrolyzed and metabolized to generate an array of relevant products (metabolites) after they enter an organism. Prototype drugs or metabolites can irreversibly bind to biological molecules to form drug/metabolite–biomolecule conjugates and be present in specific immune cells (27). They can also directly interact with major histocompatibility complex (MHC) molecules and T-cell receptors (TCRs) *via* non-covalent interactions to develop unorthodox immune responses. Accordingly, three pharmacological interaction with immune receptor (p-i) hypotheses, including altered peptide model of p-i HLA, allo-immune model of p-i HLA, and altered TCR repertoire model, have been proposed to explain how small molecule drugs or their metabolites induce immune reactions. However, not all reactions can be easily classified into these categories. In recent years, fake antigen model and drug-induced immune thrombocytopenia have been proposed to explain the role of non-covalent drug–protein interactions in DHRs on the other side (13, 28–30) **(** **Figure 2**). MHC molecules (termed HLA in humans) and TCR are essential for eliciting allergic hypersensitivity reactions from small molecule drugs.

### Hapten theory

The hapten theory is a classical explanation employed to explain the occurrence of small molecule drug ADHRs. This hypothesis states that small molecule drugs or their metabolites are too small to elicit an immune response. Instead, some of them are capable of covalently conjugating to endogenous proteins or peptides and generating antigenic drug protein adducts, which can act as antigens to evoke immune responses. These drugs or metabolites are termed as “haptens” in immunology. A distinctive characteristic of these drugs is their ability to recruit and activate B and/or T cells and induce immune responses (24). The formation of a stable covalent bond between haptens and amino acid residues in proteins is an essential step in initiating allergenicity (31). The hapten-biomolecule conjugates (drug–protein adducts or drug–peptide adducts) are introduced into antigen-presenting cells (APCs) and broken down by intracellular proteases to derive hapten–peptide fragments, which can fit the anchor position in MHC molecules and be presented to specific T cells to subsequently provoke cellular and/or humoral immune reactions (21, 27, 32). During these processes, the covalent links of the hapten–biomolecule conjugates must be stable and remain unbroken during the presentation by APCs and recognition by B- and/or T- cell receptors. The hapten theory has been widely accepted in studies on β-lactam antibiotics (31) and reactive sulfamethoxazole metabolites (33). DHRs to β-lactam antibiotics involve both immediate and non-immediate reactions, in which hapten–biomolecule conjugates are formed through nucleophilic attacks from the β-lactam antibiotics ring to the amino acid groups of the protein (34). For sulfamethoxazole, it has been proven in *in vitro* experiments that the reactive metabolite of sulfamethoxazole nitroso can perform protein conjugation and trigger DHRs (35). This type of ADHR belongs to idiosyncratic reactions that are generally independent of dosage and may occur after repeated drug administration.

### Pharmacological interaction with immune receptor theory

The p-i theory considers that small molecule drugs or their metabolites can exert their effects *via* non-covalent interactions (electrostatic interactions, hydrogen bonds, and van der Waals forces) with immune receptors (HLA or TCR) directly, triggering reversible reactions. This type of ADHR is an off-target cytotoxic reaction, which is implicated in HLA on APCs, TCR on T cells, and in the structural characteristics of drugs. These p-i responses are only restricted to unorthodox T- cell stimulation, and antibodies produced by B cells do not participate in the process (36). To date, three patterns of p-i-related hypersensitivity reactions have been proposed: the altered peptide model of p-i HLA, the allo-immune model of p-i HLA, and the p-i TCR model (24, 30). Both genetic predisposing factors and exposure to drugs affect these p-i reactions.

The altered peptide model of p-i HLA and the allo-immune model of p-i HLA were both developed because some ADRs generated by small molecule drugs are limited exclusively to the HLA allele (24, 36). Drugs themselves and specific HLA risk alleles, rather than hapten–biomolecule conjugates, may be related to adverse reactions. Common drugs and their highly restricted HLA alleles are listed in **Table 2**. The altered peptide theory speculates that when some small molecule drugs encounter APCs, they may be taken up into the endoplasmic reticulum and accidentally bind to the empty pocket on specific HLA molecules *via* non-covalent bonds. This abnormal process can change the peptide-binding ability of the involved HLA, cause alterations in the preferred peptides for binding sites on HLA, and further influence the disposal of captured peptides. As a result, when normal endogenous peptides combine with these altered HLA molecules, they can be processed into novel peptide fragments and be recognized as foreign substances by T cells, thereby leading to autoimmune reactions. Unlike the altered peptide theory, the allo-immune theory states that the whole configuration of a particular HLA complex may be changed by small molecule drugs, triggering immediate DHRs. Anchored peptides may have flexibility and are not always firmly fixed on the HLA binding groove. When these peptides move out, their interaction with HLA may be weakened, and some drug-binding sites can be partially exposed. In this instance, some small molecule drugs directly attach to the binding sites in the peptide-binding groove of HLA and form peptide–drug–HLA complexes. These complexes are immunogenic because they are similar to allo-HLA, which can cause allorecognition by T cells and trigger immunogenic responses (36, 56). The interaction between the HLA-B*5701 allele and abacavir has been confirmed, which indicates that the DHRs of abacavir may be induced by changes in the peptide repertoire of the HLA molecule (57). In addition, T- cell reactions caused by allopurinol and oxypurinol have been proven to restrict HLA-B*5801, which correlates with *in silico* docking data showing that oxypurinol binds with high affinity to the peptide-binding groove of HLA-B*5801 (44).

**Table 2:** Typical drug hypersensitivity reaction-related HLA alleles (37–39).

Drug	HLA allele	ADHRs
Abacavir (40, 41)	HLA-B*5701	Rash, gastrointestinal tract and respiratory symptoms
Allopurinol (42, 43)/Oxypurinol (metabolite of allopurinol) (44, 45)	HLA-B*5801	Maculopapular eruption, severe cutaneous adverse reactions, SJS, TEN, drug reaction with eosinophilia and systemic symptoms (DRESS)
Carbamazepine (46, 47)	HLA-B*1502 HLA-A*1301	MPE, DRESS, SJS, TEN
Methazolamide (48, 49)	HLA-B*5901 HLA-B*5502	SJS, TEN
Dapsone (50, 51)	HLA-A*1301	Eosinophilia and systemic symptoms
Oxcarbazepine (52, 53)	HLA-B*1502	SJS, TEN
Penicillin (54, 55)	HLA-B*5501 HLA-DR	DRESS, MPE, SJS, TEN

The p-i TCR model indicates that ADHRs induced by small molecule drugs are only attributed to certain limitative TCR structures. Exchangeable HLA molecules may or may not participate in these reactions. The p-i TCR theory states that in some ADHRs, small molecule drugs can interact with TCRs at some positions outside the peptide-binding sites, resulting in alterations in the configuration of TCRs. This can enhance the reactivity of T cells to peptides presented by HLA or even normal endogenous peptides. In addition, some data also show that small molecule drugs with specific structures (e.g., sulfamethoxazole with NH_2_) can directly bind to the loops of TCRs and make particular groups point to the peptide-binding groove, which directly causes T- cell activation and triggers hypersensitivity reactions. These reactions are often dose related and can be blocked by other compounds with similar chemical structures.

So far, the relationship between p-i responses and cytokines and/or costimulatory molecules that are crucial in hapten-induced allergic reactions has not been clearly expounded (24). It has been speculated that patients suffering from p-i ADRs may already undergo massive immune stimulation of T cells by other influencing factors, such as virus infection, and that they possess high level of cytokines and augmented expression of costimulatory molecules. These factors make them more likely to react to minor signals like p-i reactions (21).

### Fake antigen model

The fake antigen model proposes that after patients are sensitized by covalent drug–biomolecule conjugates, the specific IgE antibodies will bind to high-affinity FcRI on mast cells and basophils. Then, when the same drug in high concentration is re-exposed to the individuals, they may form non-covalent drug–biomolecule complexes immediately, which can bind to cross-linking specific IgE and stimulate the degranulation of mast cells and basophils in seconds or minutes. Although the non-covalent drug–biomolecule adducts cannot elicit IgE antibodies by themselves, they can quickly react with preformed IgE (30, 58).

### Drug-induced immune thrombocytopenia

Some drug-induced immune thrombocytopenia is partly due to drug-dependent antibodies, which can trigger clearance of platelets or direct platelet destruction. It has been assumed that the patients may already have low-affinity antibodies for platelet glycoprotein. When the drug is administrated, it can non-covalently bind to a complementary-determining region on the antibody, which may increase antibody affinity for a specific epitope expressed on the platelet glycoprotein. The hypothesis has been widely accepted in studies on quinine (30, 59).

## Mechanisms of non-allergic hypersensitivity reactions from small molecule drugs

**Figure 3:**
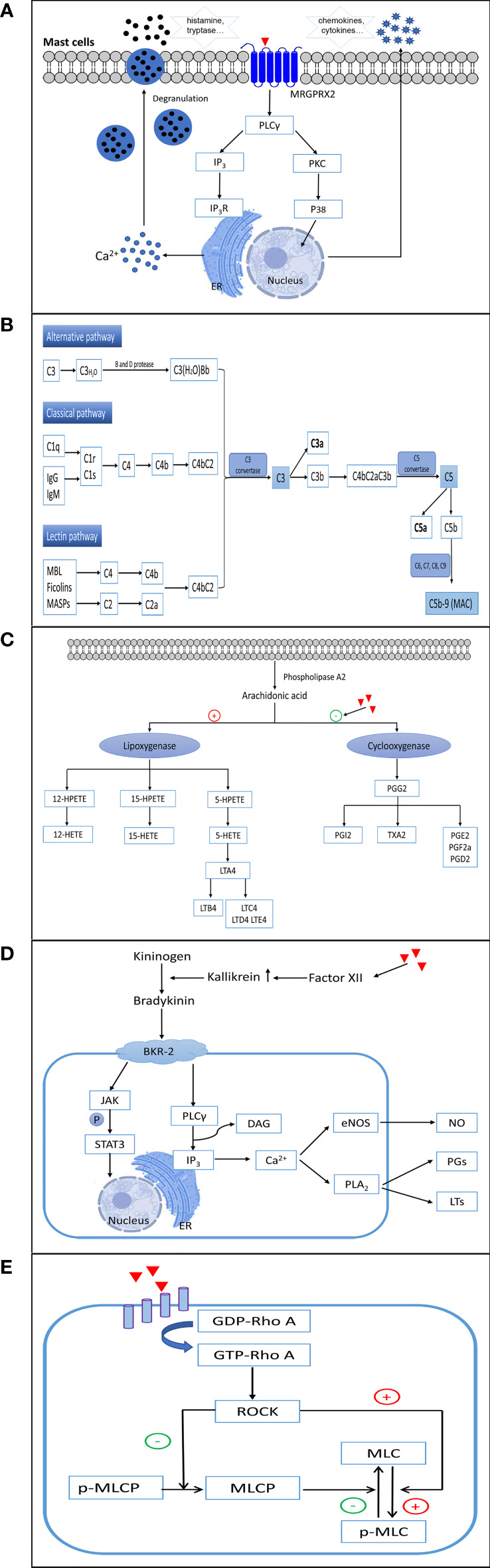
The mechanisms involved in non-allergic hypersensitivity reactions to small molecule drugs. **(A)** Direct activation of mast cells. **(B)** Activation of complement. **(C)** Inhibition of cyclooxygenases. **(D)** Elevation of bradykinin. **(E)** Activation of the RhoA/ROCK signaling pathway. ER, endoplasmic reticulum; MRGPRX2, Mas-related G protein-coupled receptor X2; PLCγ, phospholipase C gamma; IP3, inositol triphosphate; IP3R, inositol triphosphate receptor; PKC, protein kinase C; P38, p38(MAPK) pathway; MAC, membrane attack complex; 12-/15-/5-HETEs, 12-/15-/5-hydroxy eicosatetraenoic acids; 12-/15-/5-HPETE, 12-/15-/5-hydroperoxyeicosatetraenoic acid; LTA4/LTB4/LTC4/LTD4/LTE4, leukotriene A4/B4/C4/D4/E4; PGG2/PGI2/PGE2/PGE2a/PGD2, prostaglandin G2/I2/E2/E2a/D2; TXA2, thromboxane A2; BKR-2, bradykinin receptor type 2; JAK, Janus-activated kinase; STAT3, signal transducer and activator of transcription 3; DAG, diacylglycerol; eNOS, endothelial nitric oxide synthase; NO, nitric oxide; PLA2, phospholipase A2; PGs, prostaglandins; LTs, leukotrienes; GDP-Rho A, an inactive GDP-bound Ras homolog family member A; GTP-Rho A, an active GTP-bound Ras homolog family member A; ROCK, Rho-associated kinase; MLC, myosin light chain; p-MLC, phospho-myosin light chain; MLCP, myosin light chain phosphatase.

NADHRs are also termed pseudo-allergic or anaphylactoid reactions and cannot be easily distinguished from type I ADHRs. These reactions can arise at the first exposure to drugs, independent of prior sensitization, and are usually correlated with excessively high dose levels. Currently, direct activation of mast cells, provoking the complement system, stimulating or inhibiting inflammatory reaction-related enzymes, accumulating bradykinin, and/or triggering vascular hyperpermeability are considered the main factors leading to NADHRs (14, 60) **(** **Figure 3**).

### Direct activation of mast cells

Many small molecule drugs directly activate mast cells in an IgE-independent manner, which can immediately provoke the degranulation of mast cells and initiate the rapid release of multiple inflammatory mediators, such as histamine, tryptase, serotonin, heparin, and proteases. Subsequently, chemokines, cytokines, leukotriene C4 (LTC4), and prostaglandin D2 are produced, which propagate further late tissue injury (60–62). The clinical symptoms of NADHRs induced by the direct activation of mast cells mainly include rash, urticaria, angioedema, bronchospasm, diarrhea, and anaphylaxis. These reactions generally occur immediately after the first intake of the drugs, and the clinical presentations are undistinguishable from those observed in IgE-mediated ADHRs.

In humans, mast cells are classified into two types according to the proteases contained in their secretory granules. Most mast cells located in connective tissues (predominantly skin and major bronchi) containing tryptase and chymase are categorized as MC_TC_, whereas major mast cells found in the lungs and small intestine mucosa that contain tryptase, but little or no chymase, are recognized as MC_T_ (63, 64). In recent years, Mas-related G protein-coupled receptor X2 (MRGPRX2), expressed on human MC_TC_, has been discovered as a crucial receptor responsible for NADHRs. Mas-related gene (Mrg) receptors belong to class A G protein-coupled receptors, which can bind to several endogenous and exogenous ligands (65–68). To date, NADHRs produced by many drugs, such as iohexol (69), mivacurium (70), cisatracurium (71), isosalvianolic acid C (72), clozapine (73), icatibant (74), and phenothiazine antipsychotics (75), have been found to be related to MRGPRX2. Agents can bind and couple with MRGPRX2, which stimulates phosphorylation cascades, augments intracellular calcium levels, and induces degranulation of mast cells. The mitogen-activated protein kinase (MAPK), nuclear factor kappa B (NF-κB), PI3K/AKT, and PLCγ signaling pathways have been reported to correlate with MRGPRX2-mediated mast cell activation (76, 77). MrgprB2, MrgprB3, and MrgprX2, orthologs of human MRGPRX2, have been proposed as the mouse, rat, and dog mast cell basic secretagogue receptors related to non-allergic hypersensitivity reactions, respectively (68, 78–81). However, although human MRGPRX2 and MrgprB2 share certain similar characteristics, such as being selectively expressed in mast cells located in connective tissues and being activated by compound 48/80, they only share approximately 53% overall sequence identity, 34% N-terminal amino acid sequence identity, and 47% C-terminal amino acid sequence identity (68). Therefore, caution should be exercised when employing animals to test NADHRs induced by mast cell activation.

Unlike most G protein-coupled receptors, MRGPRX2 has low-affinity for agents (67). Therefore, one key factor influencing the occurrence of NADHRs *via* direct activation of mast cells is a sufficiently high drug exposure (82). Meanwhile, disease, combination medication, metabolic disorder, and individual variation can also promote the reaction between drugs and MRGPRX2. For example, patients with chronic spontaneous urticaria exhibit much stronger responses to MRGPRX2 agonists (83). NADHRs of fluoroquinolones are possibly related to delayed drug elimination (83, 84). In addition, individuals harboring missense MRGPRX2 mutations (G165E, D184H, W243R, or H259Y) have been proposed to protect NADHRs from mast cell activation, but the results have not been verified clinically (85).

### Complement activation

To mediate NADHRs, small molecule drugs with excipients may aggressively trigger the complement system (86) and result in the unusually augmented generation of toxic complement protein fragments. These non-IgE-mediated hypersensitivity reactions are related to complement activation and are commonly referred to as complement activation-related pseudo-allergy (CARPA). The exact mechanism of action of CARPA has not yet been fully elucidated. Currently, the accepted hypothesis is that agents may prompt the complement cascade by upregulating complement convertase, which results in increased production of anaphylatoxins such as C5a and C3a (87). Subsequently, anaphylatoxins bind to effector cells, such as macrophages, basophils, and mast cells, leading to the liberation of a multitude of vasoactive inflammatory mediators, including tryptase, histamine, platelet- activating factor, and leukotrienes, which continually amplify complement-induced effects (87, 88). Inflammatory mediators can continue to induce the generation of C3a and C5a to expand NADHRs (89). The process of this kind of reaction may involve the classical, alternative, and/or lectin pathways (90–92). Complement factors are present at high concentrations in the blood and tissues, which serve as extraordinarily efficient regulators to ensure that the system reacts quickly when violated by foreign agents. Among the various complement components (>30 soluble and surface-expressed proteins), C3a and C5a are recognized as the chief culprits in eliciting the degranulation and chemotaxis of mast cells and basophils *via* C3aR and C5aR on the surface of cell membranes (93, 94). Recently, it was shown that C3a-related reactions may also be implicated in PLCβ-mediated Ca^2+^ mobilization and upregulation of PKC, PI3K, and ERK. However, degranulation caused by C5a is relevant only to PLCβ (95).

NADHRs caused by radiocontrast media (96), doxorubicin (Doxil^®^) (97), paclitaxel (98), and others are likely linked to abnormal complement activation. Drug excipients such as Cremophor-EL (98), Tween-80 (99), and liposomes (100, 101) are considered to play an important role in NADHRs. However, the factors affecting these reactions remain poorly understood. In general, the primary influencing factors are exposure to the agents and the speed of administration. The risk can be decreased when the process of medication is well controlled (87, 96). In addition, the surface charge, morphological properties, size, and composition of the drugs or excipients are reported to be related to these reactions. For example, it has been proposed that ionic high-osmolarity radiocontrast media are more likely to trigger ADRs than nonionic low-osmolarity agents (96). Complement activation requires a surface that allows the deposition of complement fragments to build multimolecular C3 and/or C5 convertases and initiate the complement cascade (102, 103). Elongated and irregular liposomes with a low curvature oval and relatively long diameter of the molecules are considered to more easily activate the complement system (87, 103).

### Inhibition of cyclooxygenases

Nonsteroidal anti-inflammatory drugs (NSAIDs) comprise a heterogeneous group of compounds, including aspirin, acetylsalicylic acid, and ibuprofen. These drugs can inhibit the function of cyclooxygenases and suppress the conversion of arachidonic acids to thromboxane and prostaglandin. As a result, the augmented arachidonic acid shunt is metabolized toward the 5-lipoxygenase pathway, leading to the increased formation of leukotrienes (LTs) and cysteinyl leukotrienes (104, 105). Arachidonic acids are first transformed to 5-HPETE by 5-lipoxygenase, which is then degraded to 5-HETE and the unstable epoxide intermediate LTA4. LTA4 can then be enzymatically converted to LTB4 or combined with glutathione to generate LTC4. Subsequently, LTC4 is metabolized to sulfur-containing LTD4 and E4 leukotrienes by the sequential removal of glutamic acid and glycine. To date, four high-affinity receptors for LTs and cysteinyl leukotrienes have been identified in lung smooth muscle cells, peripheral blood leukocytes, and mast cells (BLT1 for LTB4, CysLT1 and CysLT2 for LTC4 and LTD4, and GPR99 for LTE4) (106). LTB4, C4, D4, and E4 are potent causative mediators that trigger both immediate and delayed hypersensitivity reactions and inflammation as well as provoke eosinophil chemotaxis, vascular leakage, airway remodeling, and arteriolar constriction (107–110). NADHRs resulting from the intake of NSAIDs are attributed to the indirect abnormal upregulation of leukotriene production (111, 112). An overdose of NSAIDs or metabolic disorders can result in ADRs.

### Elevation of bradykinin

Some non-allergic vascular leakages triggered by potentially harmful agents are correlated with abnormally increased bradykinin levels (113–116). The bradykinin-forming cascade is initiated by stimulation of factor XII to produce augmented kallikrein. Subsequently, high-molecular-weight kininogens are digested by kallikrein to generate bradykinin (117, 118). Bradykinin is a tissue hormone that increases vascular permeability and decreases blood pressure. The elevation of bradykinin in NADHRs mainly occurs as a result of unusual kallikrein activation and abnormal C1 inhibitor activity (113). Bradykinin B2 receptors play an essential role in the induction of angioedema. Bradykinin B2 receptors on endothelial cells can activate PLCγ, upregulate the formation of inositol 1,4,5-triphosphate and diacylglycerol, elevate intracellular Ca^2+^ levels, facilitate endothelial nitric oxide synthase, and trigger PLA2. This series of processes leads to the extraordinary generation of arachidonic acid metabolites and elevated production of prostaglandins and LTs, which cause vascular leakage (119–121). Drug-induced, bradykinin-related, non-allergic angioedema is typically defined as a side effect that is commonly observed with angiotensin-converting enzyme inhibitors (122). Patients with C1 inhibitor deficiency tend to develop bradykinin-mediated angioedema (113).

### Provocation of vascular leakage

In recent years, some small molecule drugs have been found to directly trigger aggressive vascular hyperpermeability and NADHRs (123, 124). Currently, hypersensitivity reactions caused by various traditional Chinese medicine injections (125–128), penicillin (123), and paclitaxel (124) are reported to be related to their adverse effects on vessels. So far, this opinion has been mainly proven by animal experiments, and the reactions occur in a dose-dependent manner. The RhoA/ROCK signaling pathway, which plays an essential role in regulating the cytoskeleton and endothelial barrier, is considered to be an important factor influencing these reactions (123, 124, 129).

## Screening hypersensitivity reactions in clinical practice

Due to unpredictable reactions, DHRs cannot be prevented completely before medication. In reality, a detailed clinical history is the primary clue for identifying the possibility of an allergic reaction. Moreover, many assays have also been promoted to more deeply clarify whether patients labeled as allergic are truly allergic to putative drugs and to prevent them from further exposure to the causative agents. In addition, several methods have been established to screen susceptible populations. Biomarkers, including parameters obtained from skin testing, drug-specific antibodies detected in blood, and inflammatory factors such as histamine and cytokines, are all crucial for allergic diagnosis (130). Risk HLA alleles that have been identified to show strong associations with particular drugs (such as HLA-B*5701 and abacavir) may be useful for predicting ADHRs (3, 131, 132). Currently, no diagnostic method has been specifically designed for NADHRs. These reactions can mostly be defined when clinical presentations and inflammatory factors suggest DHRs, but immune mechanisms cannot be demonstrated in the patient.

### Direct testing of specific antibodies

In clinical practice, skin testing mainly consists of prick, intradermal, and patch tests to confirm or exclude the existence of drug-specific IgE antibodies. When drug-related allergens enter the skin, they react with drug-specific IgE–effector cell complexes and cause the release of histamine and other inflammatory mediators, which results in macroscopic skin symptoms and indicates the possibility of allergy (133). Currently, this diagnostic method is mainly used for β-lactam antibiotics. Skin testing for penicillin has >95% negative predictive value, and when combined with an oral challenge, the predictive value can reach >99% (134). It is worth noting that skin testing detects the presence of allergen-specific IgE to elicit which small molecule drugs need to act as haptens to form covalent hapten–biomolecule conjugates. This method is not suitable for testing non-covalent drug–protein complexes (58).

Drug-specific IgE antibodies can be directly assessed *in vitro* using a solid phase that is functionalized with drug–carrier conjugates, followed by detection *via* fluoroimmunoassays, radioallergosorbent tests, or enzyme-linked immunosorbent assays (135, 136). During these tests, small molecule drugs and their metabolites must first bind to a carrier to obtain immunological activity, and then, drug-specific IgE in serum is quantitatively measured. In contrast to other *in vitro* antibody detection methods, frozen samples can be used to determine specific IgE levels (137). However, the complicated two-step procedure affects the sensibility and accuracy of these methods to some extent and limits their application. In addition, the basophil activation test (BAT) using flow cytometry is now widely applied as a functional assay to aid in the diagnosis of IgE-related basophil degranulation. Patients with an allergic history can undergo BAT for suspected, same class, and alternative drugs (ranging from 5 to 12 drugs) (138–141). BAT is considered to closely mimic *in vivo* type I allergic reactions with high specificity because the basophil activation percentage and mean stimulation index for CD203c expression have been found to be much higher in allergic patients in clinical practice (142, 143). Although BAT currently has up to 80% specificity, the sensitivity generally ranges between only 50% and 60%; thus, it still needs to be improved for diagnostics. At present, BAT is mainly used to investigate the immediate DHRs of antibiotics, neuromuscular blocking agents, and NSAIDs (144).

### Determination of T-cell-induced reactions

Until now, standardized methods used for the quantitative measurement of relevant parameters of T- cell recruitment and activation include enzyme-linked immune absorbent spot (ELISpot), lymphocyte transformation test (LTT), and cytokine/mediator detection assays (130, 145). ELISpot is a quantitative method that measures the parameters relevant to T- cell activation and evaluates T- cell immunity in clinical trials. In the diagnosis of DHRs, ELISpot mainly focuses on detecting cytokines of interest secreted by specific T- cell subsets in response to suspected drugs. The utility of ELISpot is highly dependent on the drug involved and biomarkers employed in the test. For example, IFN-γ can be used as a marker for CD8^+^ cytotoxic T cells, and it can distinguish different subsets of activated T cells *via* cytokines (146–148). LTT is also a relatively standard method based on evaluating the expansion of drug-specific memory T cells, which are obtained by co-incubation of the patient’s peripheral blood mononuclear cells with the suspected drug (149–151). Currently, LTT is an acceptable method applied in clinical experiments for diagnosing delayed ADHRs by detecting drug-induced T- cell proliferation. Additionally, the presence of HLA and TCR on peripheral blood mononuclear cells enables drug interaction *via* the p-i concept. Therefore, LLT may be appropriate for all DHRs correlated with T cells, regardless of the background mechanism.

### Assessment of inflammatory factors involved in NADHRs

To date, no exclusive assessment of NADHRs has been performed. As NADHRs show clinical manifestations identical to those of IgE-mediated ADHRs, some diagnostic methods focusing on inflammatory factors that are applied in ADHRs can also be used in NADHRs with minor modifications. For example, the determination of IgE-independent histamine and/or tryptase release and the evaluation of various surface markers for basophil activation (e.g., CD63 and CD203) are valuable markers for the identification of NADHRs (152). Additionally, the plasma complement terminal complex (SC5b-9) was tested to confirm the involvement of the complement system in NADHRs (97, 103).

## Hypersensitivity evaluation for investigational new drugs or post-marketing drugs

Many preclinical *in vivo* and *in vitro* tests have been conducted to evaluate the risk of drug-induced hypersensitivity reactions. However, there is currently no validated method for assessing all types of sensitizing potentials of small molecule drugs during the preclinical phase (153). In the FDA guidance on immunotoxicology evaluation, passive cutaneous anaphylaxis, active cutaneous anaphylaxis, and active systemic anaphylaxis assays have been used to predict type I reactions. Although these assays have been used to detect allergenic proteins, they have not been proven to be effective enough to determine the adverse effects of small molecule drugs because the production of the underlying haptens cannot be considered. Positive reactions in the assays may indicate that the drug has sensitizing potential, whereas negative results cannot confirm a lack of immunization. At the same time, the Buehler test, guinea pig maximization test, and murine local lymph node assay (LLNA) have been recommended by the Center for Drug Evaluation and Research to evaluate the topical sensitizing potential of drugs. These methods are considered reliable and have a high correlation with known human skin sensitizers, which can be used to evaluate the safety of investigational new drugs to support clinical trials. Detailed guidelines for the Buehler test, guinea pig maximization test, and LLNA are also recommended by the Organization for Economic Co-operation and Development (OECD). In addition, the lymph node proliferation assay, a modified LLNA method, has been reported to further predict systemic hypersensitivity (154, 155). Moreover, mechanistically based *in chemico* skin sensitization assays addressing the key event of the adverse outcome pathway (AOP) on covalent binding to proteins, *in vitro* skin sensitization assays addressing the AOP key event on keratinocyte activation, and the key event in the activation of dendritic cells on the AOP for skin sensitization are also issued by the OECD for risk assessment. All three methods have been reported to possess high sensitivity and specificity. The three methods with LLNA build an evaluation system for the skin sensitization AOP. In addition to the methods recommended by the FDA and OECD, high-throughput and standardized BAT and LTT methods can quickly deliver information to hint at the possibility of ADHRs induced by drug-specific IgE or T cells, which could be acceptable in preclinical hypersensitivity testing. Additionally, the ability of drugs to bind to HLA and/or TCR *via* non-covalent interactions has been widely investigated using peripheral blood mononuclear cell stimulation in combination with molecular docking (156).

For NADHRs, experiments focusing on abnormal histamine release, vascular leakage, and complement activation have been established. MrgprB2^MUT^ mice (79) and mouse vascular permeability evaluation models (123, 125) have been developed to investigate these underlying mechanisms. In addition, animal models using different species to mimic the responses in complement activation-related NADHRs have also been developed (157, 158). Instead of prediction, these methods are mostly used to investigate the underlying mechanisms involved in existing ADRs. Furthermore, some *in vitro* methods focused on mast cell degranulation and histamine/β-hexosaminidase release, complement system activation, changes in intracellular Ca^2+^ levels, and alterations in the endothelial cytoskeleton and monolayer permeability have been applied to predict the potential of drugs to induce NADHRs (99, 123, 129, 159, 160). However, the drug concentrations used in *in vitro* experiments still need to be optimized because NADHRs are highly correlated with drug exposure.

## Conclusion

As unsolved and costly public health issues, hypersensitivity reactions to small molecule drugs have attracted widespread attention. However, the mechanisms involved in allergic and non-allergic reactions have not yet been fully clarified and verified. Advanced, highly effective, and sensitive prediction methods for clinical and non-clinical investigations are urgently needed.

## Author contributions

JH, QL, and YZ are the major writers of the manuscript. CP and XT have drawn the pictures. YSZ and AL have overseen the writing. All authors contributed to the article and approved the submitted version.

## Funding

This work was supported by grants from Beijing Natural Science Foundation (7214291), National Natural Science Foundation of China (82104519 and 82192913), CACMS Innovation Fund (CI2021A04803 and CI2021B016), CACMS Foundation (ZZ15-YQ-045), and Fundamental Research Funds -for the Central Public Welfare Research Institutes (ZXKT21018).

## Conflict of interest

The authors declare that the research was conducted in the absence of any commercial or financial relationships that could be construed as a potential conflict of interest.

## Publisher’s note

All claims expressed in this article are solely those of the authors and do not necessarily represent those of their affiliated organizations, or those of the publisher, the editors and the reviewers. Any product that may be evaluated in this article, or claim that may be made by its manufacturer, is not guaranteed or endorsed by the publisher.

## Abbreviations

ADHRs, allergic drug hypersensitivity reactions; ADRs, adverse drug reactions; APCs, antigen-presenting cells; AOP, adverse outcome pathway; BAT, basophil activation test; DHRs, drug hypersensitivity reactions; ELISpot, enzyme-linked immune absorbent spot; HLA, human leukocyte antigen; IgE, immunoglobulin E; IgG, immunoglobulin G; IgM, immunoglobulin M; LTs, leukotrienes; LTT, lymphocyte transformation test; LLNA, local lymph node assay; MHC, major histocompatibility complex; MRGPRX2, Mas-related G protein-coupled receptor X2; NSAIDs, nonsteroidal anti-inflammatory drugs; NADHRs, non-allergic drug hypersensitivity reactions; TCR, T-cell receptor.
